# Effects of intravenous tranexamic acid on ovarian reserve and intra-operative blood loss during laparoscopic cystectomy of endometriotic cyst: a pilot randomized controlled trial

**DOI:** 10.1186/s40814-021-00907-y

**Published:** 2021-09-04

**Authors:** Prangthip Akkaranurakkul, Srithean Lertvikool, Woradej Hongsakorn, Orawin Vallibhakara, Siriluk Tantanavipas, Krissada Paiwattananupant, Wichai Ittichaikulthol, Apirom Vongsakulyanon, Sakda Arj-Ong Vallibhakara, Makaramas Anantaburana, Areepan Sophonsritsuk

**Affiliations:** 1 Department of Obstetrics and Gynaecology, Faculty of Medicine Ramathibodi Hospital, Mahidol University, Bangkok, 10400 Thailand; 2 Reproductive Endocrinology and Infertility Unit, Department of Obstetrics and Gynaecology, Faculty of Medicine Ramathidodi Hospital, Mahidol University, Praram 6 Rd., Phayatai, Bangkok, 10400 Thailand; 3 Gynecologic Oncology Unit, Department of Obstetrics and Gynaecology, Faculty of Medicine Ramathidodi Hospital, Mahidol University, Bangkok, 10400 Thailand; 4 Department of Anesthesiology, Faculty of Medicine Ramathidodi Hospital, Mahidol University, Bangkok, 10400 Thailand; 5 Department of Pathology, Faculty of Medicine Ramathibodi Hospital, Mahidol University, Bangkok, 10400 Thailand; 6 ASEAN Institute for Health Development, Mahidol University, Nakorn Pathom, 73170 Thailand

**Keywords:** Anti-fibrinolytic Agents, Anti-Mullerian hormone, Endometriosis, Ovarian reserve, Tranexamic acid

## Abstract

**Background:**

Strategies to preserve ovarian function after ovarian endometriotic cyst removal have been reported in many studies; however, no study has evaluated tranexamic acid administration during surgery.

**Objective:**

To evaluate feasibility of conducting a definitive trial and assessing the potential efficacy of tranexamic acid on ovarian reserve and intra-operative blood loss by comparing mean differences in anti-Müllerian hormone (AMH) levels following laparoscopic ovarian cystectomy between tranexamic acid and control groups.

**Materials and methods:**

A parallel two-arm pilot trial was conducted with 40 participants with endometriotic cysts who underwent laparoscopic ovarian cystectomy. They were randomized 1:1 to either 1 g tranexamic acid (TXA) or no TXA (*n* = 20 per group). TXA was administered to the participants immediately after induction of general anesthesia and intubation. The primary outcome was the feasibility of conducting a definitive trial in terms of design and procedures (such as recruitment rate, retention, safety of intravenous 1 gm of TXA, sample size verification) and assess the efficacy of TXA on the ovarian reserve and intra-operative blood loss by comparing mean difference of AMH levels between TXA and control groups at pre- and 3 months post-surgery.

**Results:**

The recruitment and successful completion rates were 95% and 100%. Baseline characteristics were similar in the two groups. The mean difference of serum AMH levels (pre- and 3 months post-surgery) between the TXA and control groups was not significantly different. When performing a subgroup analysis, the mean difference of AMH levels (pre- and 3 months post-surgery) seemed to be higher in the bilateral than in the unilateral ovarian cyst group but not significantly different. Operating time was significantly longer in bilateral than in unilateral cysts. No post-operative complications or adverse effects were found.

**Conclusion:**

The full randomized controlled trial for evaluating effects of TXA administration during laparoscopic cystectomy for endometrioma on ovarian reserve was shown to be feasible. Several modifications should be added for improving feasibility, for example, increasing the TXA dose, modifying TXA administration, focusing on either patients with unilateral or bilateral ovarian cysts, and exploring other outcome measures, e.g., surgeons’ satisfaction.

**Trial registration:**

Thai Clinical Trials Registry, TCTR20190424002, Registered 24 April 2019.

## Key messages regarding feasibility

- What uncertainties existed regarding the feasibility of this study?
  Uncertainty regarding recruitment and retention of patients who have ovarian endometriotic cysts undergoing laparoscopic cystectomy and administration of tranexamic acid to preserve ovarian function.
  Uncertainty regarding the ability of patients to complete the study.
- What are the key feasibility findings?
  The recruitment and retention of patients was feasible and showed zero rate of incompleteness of the study
- What are the implications of the feasibility findings for the design of the main study?
  Modifications of study design would be needed for conducting the full randomized controlled trial. Many suggestions for the next study include a probable increase in the dosage of tranexamic acid, a study on similar patients with either unilateral or bilateral ovarian cyst, and investigation of other outcome variables.

## Introduction

Ovarian endometriosis (endometriomas) is a common gynecological disease that occurs up to 10% of reproductive women and the prevalence of disease is up to 20 to 50% in infertile women [1–3]. The clinical presentations include pelvic pain, progressive dysmenorrhea, dyspareunia, and subfertility. The presence of endometriomas has the potential to destroy healthy ovarian tissues that leads to decreased ovarian function [4], problems with ovulation [5], and primary ovarian insufficiency [6]. The most effective treatment for endometriotic cyst is controversial. The first-line management of endometriotic cyst, a diameter larger than 3 cm, is a laparoscopic ovarian cystectomy [7]. Moreover, management of endometrioma larger than 3 cm in women who have an infertility problem should consist of ovarian cystectomy prior to assisted reproductive technologies to improve pelvic pain or help perform the oocyte retrieval procedure without difficulties [8]. Although laparoscopic ovarian cystectomy provides the lowest recurrence and the highest chance of spontaneous pregnancy rate, risk of significant ovarian injury can occur. Ovarian injury is thought to be caused by loss of healthy ovarian follicles during surgery and inflammation caused by surgical trauma or vascular injury. Recent data have demonstrated that surgical treatment of endometriotic cyst have an adverse effect on ovarian reserve [9]. Inadvertent removal of normal ovarian tissue is one of the reasons for the reduction in ovarian reserve during cystectomy [10]. Serum anti-Müllerian hormone (AMH) is the most reliable and practical measurable marker for ovarian reserve [11]. This hormone reflects the number of high-quality oocytes within the ovaries. AMH is secreted by granulosa cells in women of reproductive age. Several studies have demonstrated a decrease in AMH levels after laparoscopic cystectomy of endometriomas [12–14]. In our study, AMH was used to evaluate ovarian reserve. The advantage of AMH over other ovarian serum markers is fairly constant levels, and hormone levels can be measured on any day of the menstrual cycle. AMH levels are reliable for predicting fertility and helping physicians identify women at risk of premature ovarian insufficiency [15, 16].

Many gynecologists try to find strategies to preserve ovarian function. We hypothesized that if a decrease in blood loss during surgery occurs, the surgeon could view the operative field clearly and could perform the operation smoothly with less use of bipolar coagulation. As a result, vascular and healthy ovarian tissue damage would be decreased. In the present study, we use tranexamic acid (TXA) as a pharmacological tool for reducing bleeding during surgery. TXA has been widely used during surgery to decrease bleeding and wound complications [17–19]. The recommended standard dose is 1 g every 6 to 8 h in general fibrinolysis [20]. However, the dose could be increased in case of excessive bleeding but should not exceed a maximum daily dose of 4 g [21].

The objectives of this study were to evaluate the feasibility of conducting a definitive trial in terms of design and procedures (such as recruitment rate, retention, safety of intravenous 1 g of TXA, and sample size verification) and assess TXA efficacy on ovarian reserve and intra-operative blood loss by comparing the mean difference of AMH levels between TXA and control groups. AMH was measured pre- and at 3 months post-surgery.

## Materials and methods

This study was a double-blind randomized controlled trial (RCT) conducted between May 2019 and November 2020. The study was approved by the Ethical Clearance Committee on Human Related to Researches Involving Human Subjects and Faulty of Medicine Ramathibodi Hospital Mahidol University (No. MURA2019/245) and was registered on the Thai Clinical Trial Registry (TCTR). This study was conducted in accordance with the 1964 Helsinki Declaration.

Reproductive women ages 19–45 years who were planning to undergo laparoscopic cystectomy for unilateral or bilateral endometriotic cysts were invited to participate in the study. Inclusion criteria included several parameters: (1) unilateral/bilateral endometriotic cyst with typical ultrasonography characteristics [22], (2) ovarian cyst size 3–10 cm, (3) no previous use of oral hormones 3 months prior to the study, (4) no history of taking depot-medroxyprogesterone acetate or gonadotropin releasing hormone agonist within 9 months prior to the study, and (5) willingness to participate in the study. Exclusion criteria included several parameters: (1) pregnancy and post-menopausal status, (2) history of allergy or contraindications to TXA, (3) underlying disease, such as thromboembolic disease, which contradicts the use of TXA, (4) pre-operative AMH level < 0.5 ng/ml, and/or (5) history of previous ovarian surgical intervention. Eligible participants were enrolled in the present study and signed the consent forms. The participants were randomly assigned either: (1) TXA and (2) untreated or control. Blocks of four were randomized with a 1:1 ratio by an independent nurse and concealed in a labeled envelope. Serum samples were collected before laparoscopic ovarian cystectomy. For the intervention group, TXA 1 g intravenously was administered by anesthesiologists within 10 min before making skin incision. Surgeons were blinded to the participant groups. Laparoscopic surgery was performed with the standard operating procedure by the endoscopic staff in the Ramathibodi hospital. All surgeons (*n* = 5) had comparable surgical skills and experience with laparoscopy. Briefly, after general anesthesia was administered, the participants laid in the Trendelenburg position. The primary trocar was placed at the umbilicus with 2 to 3 accessory ports. Ovarian cystectomy was initiated by an incision over the wall of ovarian cyst through the cortex. The cyst wall was mobilized by sharp and blunt dissection and removed from the ovarian cortex. The inner ovarian stroma was coagulated with bipolar electrocautery (20–30 W current) to achieve satisfactory hemostasis and approximation. The ovarian cortex was closed with an absorbable suture (2–0 or 3–0). Blood loss was calculated by anesthesiologists as the difference between the total amounts of suction and irrigation. All intra- and post-operative complications occurring within 3 months of the operation were recorded. The participants were scheduled for follow-up appointments at 3 months post-surgery. Sera were collected for measurement of post-surgery AMH levels.

### Outcomes

The primary objective of this study was to evaluate the feasibility of conducting a definitive trial in terms of several parameters: (1) recruitment rate, (2) successful completion of the study procedures, (3) safety of intravenous 1 g of TXA, and (4) verification of the sample size calculation for the full RCT using intravenous 1 g of TXA on ovarian function preservation in patient who undergoing ovarian cystectomy. Recruitment and successful completion rate observed in the pilot which are considered feasible for the full RCT were not less than 90% of all eligible patients and 95% of all enrolled patients, respectively. Safety of intravenous 1 g of TXA is defined as no serious adverse drug reaction (ADR). Serious ADR denoted as any unfavorable medical occurrence that at 1 g TXA: (i) resulted in death; or (ii) was life-threatening; or (iii) prolonged hospital stay; or (iv) resulted in significant handicap; or (v) required medical or surgical intervention to prevent a permanent defect of a body function or structure; or (vi) was a malignancy or a congenital anomaly [23].

The secondary objectives included assessments of TXA efficacy on ovarian reserve and intra-operative blood loss by comparing mean difference of AMH levels between TXA and control groups. AMH was assessed pre-operatively and at 3 months post-surgery. AMH was quantified using an electrochemiluminescence assay (ECLIA; Elecsys® AMH assay, Roche Diagnostics) by a technician who was unaware of participant allocation.

### Sample size

The intended sample size for the present study had been estimated using a formula calculated based on the probability of observing problems occurring in target study subjects with a chosen level of confidence. *P*(*x* > 0) = 1 – (1 − *π*)^n^. Where *x* indicates as number of participants (of the *n* participant). The formula is: *n* = ln(1 − γ)/ln(1 − π) if π designates the problem probability and γ denotes certain threshold of confidence by 100% × γ (such as 95% CI, γ is 0.95) [24, 25]. The calculator is available at http://www.pilotsamplesize.com).

The lowest recruitment rate approximately 5–10% with at 95% confidence interval was used to calculate for the present study. Twenty-eight to 58 participants were needed for the unsuccessful recruitment rate of 5–10%. We chose 40 participants to enroll in the present study and estimated that it would require 15 months, including a 3-month follow-up, to complete the study.

### Statistical methods

Software program SPSS version 21.0 (SPSS Inc., Chicago, USA) was used for statistical analysis. The Pearson’s chi-square and Student’s *t* tests were applied to compare categorical and continuous parametric data, respectively. The Mann–Whitney test was used to compare nonparametric continuous data, that is, serum AMH levels before and after surgery and the difference in AMH levels (pre- and 3-month post-surgery). *P* values < 0.05 were considered to be statistically significant. The median difference and 95% confidence interval (CI) of difference were calculated by Stata Statistical Software: Release 15.0 (College Station, TX, USA) and Hodges-Lehmann median methods. (https://www.real-statistics.com/non-parametric-tests/mann-whitney-test/mann-whitney-median-confidence-interval/)

## Results

### Recruitment, recruitment rate, and the successful completion of the study procedures

**Fig. 1:**
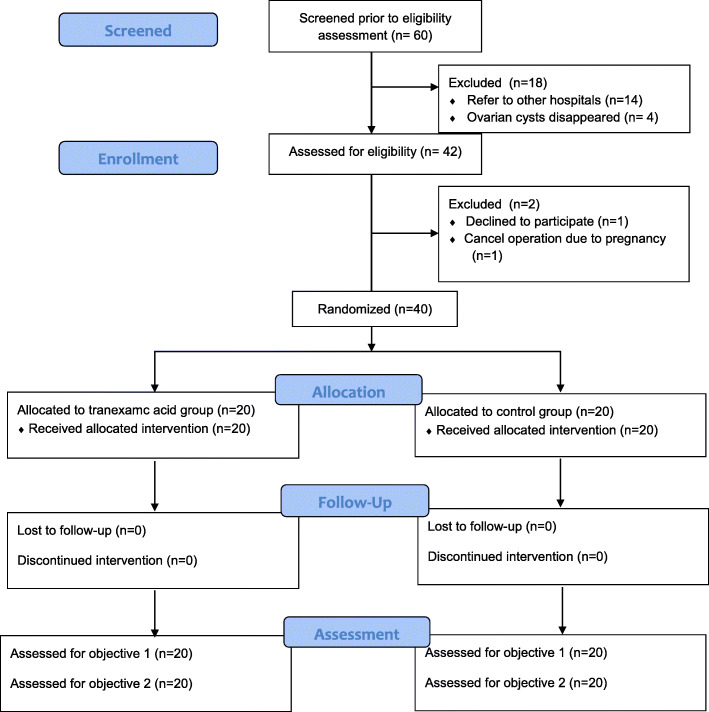
Flow chart of the study

Enrollment took place from May 2019 through August 2020, and the 3-month follow-up was completed in November 2020. The timeline for recruitment was delayed by 4 months because of the start of the coronavirus 2019 (COVID-19) pandemic in Thailand. The operations for benign conditions, which included laparoscopic cystectomy, were temporarily halted for 4 months in our hospital. Of the total 42 participants who were invited to participate in this study, 40 participants were recruited and randomized into two groups. The recruitment rate was 95%. All of them completed a 3-month follow-up, as shown in Fig. 1. We made a phone call to the participants if they missed the follow-up day and invited them to visit on the next day. The rate of successful completion of the study procedures was 100%.

### Baseline and outcome characteristics

The baseline demographic characteristics of the participants are presented in Table 1. The two groups were similar in mean age, body mass index, parity, the maximum diameter of ovarian cyst, laterality of lesions, and American Society of Reproductive Medicine (ASRM) stage of endometriotic cyst. The surgical outcomes were compared in treatment and control groups. Estimated blood loss, post-operative complications, and length of hospital stay were all similar in the two groups. However, operating time in TXA group was longer than in the control group. Pre-operative AMH levels were also similar between patients in the TXA and control groups (2.9 ± 1.5 and 2.4 ± 1.8 ng/ml, respectively) as shown in Table 2. At 3 months post-surgery, serum AMH levels of the TXA and control groups decreased significantly. Means difference of pre- and 3-month post-operative AMH of TXA and control groups were 0.89 (95% CI 0.39 to 1.38) ng/ml and 0.83 (95% CI 0.30 to 1.37) ng/ml, respectively (Table 3). However, the mean difference of serum AMH level (pre-and 3-month post-surgery) between the TXA and control groups was not significantly different (0.05; 95% CI − 0.65 to 0.76). The mean difference in serum AMH level (pre-and 3-month post-surgery) was 0.7 ± 1.2 and 0.8 ± 1.0 ng/ml in the control and TXA groups, respectively (Table 2). No post-operative complications or adverse effects were found, and none of the participants required a blood transfusion.

**Table 1:** Baseline characteristics

Characteristics	Tranexamic acid (***n*** = 20) mean **+** SD or ***n*** (%)	Control (***n*** = 20) mean **+** SD or ***n*** (%)
Age (years)	30.6 ± 4.6	32.3 ± 4.9
Body mass index (kg/m^2^)	20.2 ± 2.5	20.6 ± 3.7
Parity, *n* (%)		
Nulliparous	19 (95%)	19 (95%)
Parous	1 (5%)	1 (5%)
Maximum diameter of ovarian cyst (cm)	5.8 ± 1.8	5.4 ± 1.4
Number of ovarian cyst side, *n* (%)		
Unilateral	14 (70%)	12 (60%)
Bilateral	6 (30%)	8 (40%)
ASRM stage, *n* (%)		
Stage 3	15 (75%)	11 (55%)
Stage 4	5 (25%)	9 (45%)

*Note*: *ASRM* American Society of Reproductive Medicine, *SD* standard deviation

**Table 2:** Outcomes between treatment and control groups

	Tranexamic acid (***n*** = 20) mean **+** SD or median (IQR)	Control (***n*** = 20) mean **+** SD or median (IQR)	Mean or median difference	95% CI
Pre-operative AMH (ng/ml)	2.9 ± 1.5	2.4 ± 1.8	0.50	− 0.56 to 1.56
Post-operative AMH (ng/ml)	2.0 ± 1.5	1.6 ± 1.1	0.40	− 0.44 to 1.24
Difference of AMH level (ng/ml) ^a^	0.8 ± 1.0	0.7 ± 1.2	0.10	− 0.61 to 0.81
Operative time (min)	130.0 (82.5)	120 (106.3)	0	− 35.00 to − 30.00
Estimated blood loss (ml)	50.0 (50.0)	65.0 (150)	0	− 50.00 to 0
Length of hospital stay (days)	1.8 ± 0.4	1.9 ± 0.4	− 0.10	− 0.36 to 0.16

*Note*: *AMH* anti-Müllerian hormone, *IQR* interquartile range, *SD* standard deviation, *CI* confidence interval

^a^Difference of AMH level = pre-operative AMH-post-operative AMH

*The test of significance for pilot trials is not helpful as such studies are not designed for statistical significance

**Table 3:** Outcomes between pre- and post-operative AMH levels

	Pre-operative AMH mean **+** SD (ng/ml) (***n*** = 20)	Post-operative AMH mean **+** SD (ng/ml) (***n*** = 20)	Mean difference	95% CI
Tranexamic acid	2.9 ± 1.5	2.0 ± 1.5	0.90	− 0.06 to 1.86
Control	2.4 ± 1.8	1.6 ± 1.4	0.80	− 0.23 to 1.83

*Note*: *AMH* anti-Müllerian hormone, *SD* standard deviation, *CI* confidence interval

*The test of significance for pilot trials is not helpful as such studies are not designed for statistical significance

In our study, we analyzed the outcomes between unilateral and bilateral ovarian cysts. There were no significant differences in the mean difference of AMH levels (pre- and 3-month post-operation) between unilateral and bilateral ovarian cysts (− 0.61; 95%CI − 1.32 to 0.11). Moreover, operating time was significantly longer in patients with bilateral ovarian cysts than those with unilateral ovarian cysts, as shown in Table 4. We also analyzed subgroups consisting of pre- and 3-month post-surgery AMH levels by comparing control and TXA groups for each unilateral and bilateral ovarian cyst group. The mean difference of AMH levels in the TXA and control groups in women with unilateral ovarian cyst was not significantly different (0.06, 95%CI − 0.86 to 0.98). Operating time and blood loss were not significantly different between TXA and control groups as shown in Table 5. The mean difference in AMH levels in the TXA and control groups in women with bilateral ovarian cyst was not significantly different (0.22, 95%CI − 0.98 to 1.42). Operating times and blood loss were also not significantly different between TXA and control groups as shown in Table 6.

**Table 4:** Outcomes between women with bilateral and unilateral ovarian cysts

	Unilateral ovarian cyst (***n*** = 26) mean **+** SD or median (IQR)	Bilateral ovarian cyst (***n*** = 14) mean **+** SD or median (IQR)	Mean or median 0difference	95% CI
Difference of AMH level (ng/ml)^a^	0.5 ± 1.1	1.2 ± 1.0	− 0.7	− 1.41 to 0.01
Estimated blood loss (ml)	50 (35)	100 (150)	− 50.00	− 100.00 to 0
Length of hospital stay (days)	2 (0)	1.9 (0)	0.00	0 to 0
Operation time (min)	124.4 ± 38.8	181.6 ± 71.3	− 57.2	− 92.26 to − 22.14

*Note*: *AMH* anti-Müllerian hormone, *SD* standard deviation, *CI* confidence interval

^a^Difference of AMH level = pre-operative AMH-post-operative AMH

*The test of significance for pilot trials is not helpful as such studies are not designed for statistical significance

**Table 5:** Outcomes between treatment and control group in women with unilateral ovarian cyst

	Tranexamic acid (***n*** = 14) mean **+** SD or median (IQR)	Control (***n*** = 12) mean **+** SD or median (IQR)	Mean or median difference	95% CI
Difference of AMH level (ng/ml)^a^	0.6 ± 1.0	0.5 ± 1.2	0.10	− 0.79 to 0.99
Estimated blood loss (ml)	50 (18)	77.5 (60.8)	0	− 30.00 to 10.00
Length of hospital stay (days)	2 (0)	2 (0)	0	0 to 0
Operation time (min)	131.0 ± 38.0	116.7 ± 39.9	14.30	− 17.27 to 45.97

*Note*: *AMH* anti-Müllerian hormone, *SD* standard deviation, *CI* confidence interval

^a^Difference of AMH level = pre-operative AMH-post-operative AMH

*The test of significance for pilot trials is not helpful as such studies are not designed for statistical significance

**Table 6:** Outcomes between treatment and control groups in women with bilateral ovarian cysts

	Tranexamic acid (***n*** = 6) mean **+** SD or median (IQR)	Control (***n*** = 8) mean **+** SD or median (IQR)	Mean or median difference	95% CI
Difference of AMH level (ng/ml)^a^	1.3 ± 1.0	1.1 ± 1.0	0.22	− 0.98 to 1.42
Estimated blood loss (ml)	100 (75)	150 (175)	− 50.00	− 150.00 to 50.00
Length of hospital stay (days)	2 (0)	2 (0)	0.00	− 1.00 to 1.00
Operation time (min)^a^	156.7 ± 60.9	200.4 ± 76.5	− 43.71	− 126.58 to 39.16

*Note*: *AMH* anti-Müllerian hormone, *SD* standard deviation, *CI* confidence interval

^a^Difference of AMH level = pre-operative AMH-post-operative AMH

*The test of significance for pilot trials is not helpful as such studies are not designed for statistical significance

### Verification of sample size calculation for the full RCT

We then performed the statistical calculation for power from our results using the mean difference between pre- and post-surgery AMH levels and SD for TXA and control groups were 0.8 ± 0.1 and 0.7 ± 1.2, respectively. The power was only 4.2% (www.openepi.com). We also calculated the sample size for future full-scale randomized control studies addressing the effects of TXA on ovarian reserve during laparoscopic ovarian cystectomy for endometrioma with the formula comparing two means. Our revised sample size calculation verified 1915 experimental and 1915 control participants to allow for rejection of the null hypothesis with probability (power) 0.8 and type I error probability of 0.05. With a 10% allowance for dropouts, the total sample size would be 4214.

## Discussion

The present pilot study demonstrated that conducting a large randomized double-blind controlled trial would be feasible with some modifications. Our results do not rule out the benefit of the administration of intravenous TXA immediately before laparoscopic cystectomy in patients with endometriotic cysts. TXA seems beneficial for blood loss when we analyzed subgroups of unilateral and bilateral ovarian cyst patients. However, several modifications should be made to improve feasibility, such as increasing the TXA dosage, modifying TXA administration, studying women either with unilateral or bilateral ovarian cysts, increasing the follow-up interval, and examining other outcomes, e.g., ease of surgery and surgeon’s satisfaction.

The present study’s recruitment and retention rate was high because TXA administration is quite a safe intervention and could be theoretically beneficial to patients. The follow-up time after surgery is short, and follow-up visits are often needed to prevent the recurrence of the disease. These factors could increase the willingness of patients to come back for follow-up appointments. The delay in recruitment in the present study was unavoidable because of the global pandemic.

The laparoscopic cystectomy for endometriotic cysts caused a decrease in ovarian reserve at 3 months after surgery. Results from our study were similar to the others [26–28]. The proposed mechanisms for decreasing ovarian reserve include inadvertent removal of normal ovarian tissue during cystectomy and thermal injury. Ovarian parenchymal tissue and primordial follicle of normal ovarian tissue were observed in the specimens collected from the surgeries [29, 30]. Electrocauterization, such as bipolar electrosurgery, which is often used to control bleeding during laparoscopic cystectomy, could damage ovarian follicles [31].

Several strategies have been reported to prevent ovarian reserve decline after laparoscopic ovarian surgery, including different surgical techniques, chemical agents, and medications [32–34]. Ovarian cystectomy reduced ovarian reserve after surgery more than ovarian ablation (or vaporization) and deroofing; however, cyst recurrence was found more often in patients treated with ovarian ablation and deroofing than cystectomy [26, 35]. A systematic review involving 1047 patients demonstrated that laparoscopic ovarian suture preserved ovarian function more than bipolar electrosurgery did. A hemostatic sealant agent was superior to bipolar coagulation. Ultrasonic electrosurgery was equal to bipolar coagulation [36]. However, studies of the effect of TXA on ovarian reserve in laparoscopic cystectomy, have not been previously reported.

TXA is widely used to decrease blood loss in many situations. TXA (trans-4-(Aminomethyl) cyclohexanecarboxylic acid) is a synthetic derivative of the amino acid lysine that competitively inhibits the activation of plasminogen to plasmin and is a competitive inhibitor of tissue plasminogen activator. It inhibits the lysine-binding sites of plasminogen, resulting in inhibition of plasminogen activation and fibrin binding to plasminogen and, therefore, leads to impairment of fibrinolysis. High doses of TXA reduce plasmin formation [37]. Many strong pieces of evidence demonstrate that TXA causes a reduction in blood loss during major surgery. A large systematic review of several RCTs in 10,488 surgical patients comparing TXA/no TXA administration (placebo) demonstrated that TXA contributes to a reduction in approximately one-third of blood transfusion requirements [19]. The dosage of TXA for local fibrinolysis treatment is 0.5 to 1 g (equivalent to 15 mg/kg) by intravenous injection every 6 to 8 h while the dosage for general fibrinolysis treatment is a single dose of 1 g or 10 mg/kg by intravenous injection [38]. The meta-analysis by Heyns et al. suggested that the most frequently used single dose for a reduction of perioperative estimated blood loss in several types of operation was 15 mg/kg [39]. A study by Abbasi et al. compared two doses of TXA, i.e., 5 and 15 mg/kg during sinus endoscopy surgery [40]. The study demonstrated that the administration of TXA 15 mg/kg reduced more blood loss and more surgeons satisfied surgical field than those of TXA 5 mg/kg. Therefore, the single dose of TXA 1 g (equivalent to 15 to 20 mg/kg) was selected to explore in the present study. However, a single dose of TXA up to 100 mg/kg had been reported in coronary artery surgery [41].

The present study did not demonstrate any beneficial effect of TXA for reducing blood loss. Blood loss from the use TXA was less than that in control but not significantly different (50 vs 60 ml). The possible explanation for the different results when compared to the previous meta-analysis could be the smaller amount of blood loss in the present study. Because the amount of blood loss was small (only 50–60 ml), consequently, the difference of amount blood loss between TXA and the control group was even smaller and difficult to assess precisely. Moreover, the laterality of the ovarian cyst could have interfered with the results. In general, patients with unilateral ovarian cystectomy showed less blood loss during surgery than bilateral procedures.

Our study was the first report to evaluate the benefit of TXA on ovarian reserve, which administered before laparoscopic ovarian cystectomy by measured mean difference AMH level preoperative and 3 months after surgery. We did not find that intra-operative TXA can help preserve ovarian function. Although the sample size of the present study was small, our revised sample size calculation verified 4214 participants to allow for rejection of the null hypothesis. Therefore, it is very unlikely that future study could be conducted with an enormous sample size, even studying in multi-centers.

Ovarian laterality is a significant factor impacting ovarian reserve in patients undergoing cystectomy. Based on previous studies, bilateral cystectomy was statistically associated with a significant reduction in AMH levels and ovarian reserve compared to the unilateral cystectomy group [28, 42]. However, our study demonstrated a decrease in serum AMH levels post-surgery more in the bilateral than in the unilateral cysts, but these results did not present a significant difference. This finding could have occurred because the sample size was not calculated according to laterality. Few side effects of TXA have ever been reported [37], which is consistent with our study.

The strengths of the study were evaluated. Our study was a pioneer and a double-blinded RCT study, and surgeons with the same experience levels performed the surgery. Our study had limitations, including short-term follow-up and no assessment of other ovarian reserve markers (follicle-stimulating hormone [FSH], inhibin-B) or sonographic markers. The present study results will help guide future studies of ovarian reserve reduction prevention during ovarian surgery in terms of optimal dosage and administration methods of tranexamic acid, laterality of ovarian cysts, and other outcome variables, e.g., surgeons’ satisfaction and ease of the operation.

## Conclusions

The results from the present study support the feasibility of conducting the full RCT for the intravenous TXA administration during laparoscopic cystectomy for endometrioma. Several modifications should be added to achieve the full RCT, such as increasing the TXA dose, focusing on patient subgroups (either with unilateral or bilateral ovarian cysts), exploring surgeons’ satisfaction, and follow-up periods longer than 3 months.

## Data Availability

The datasets generated and/or analyzed during the current study are available from the corresponding author on request.

## Abbreviations

ASRM: American Society of Reproductive Medicine
AMH: Anti-Müllerian hormone
CI: Confidence interval
COVID-19: Coronavirus 2019
ECLIA: Electrochemiluminescence assay
FSH: Follicle stimulating hormone
PPH: Post-partum hemorrhage
RCT: Random controlled trial
STD: Standard division
TXA: Tranexamic acid
TCTR: Thai Clinical Trial Registry
